# A Bill—Entitled an Act to Repeal and Re-Enact with Amendments Article Thirty-Two, of the Code of Public General Laws, Entitled "Dentistry."

**Published:** 1894-04

**Authors:** 


### Editorial, Etc.

THE following is the dental bill passed by the General Assembly of Maryland April, 1894, but not approved by Governor Brown:

A BILL—ENTITLED AN ACT TO REPEAL AND RE-ENACT WITH AMENDMENTS ARTICLE THIRTY-TWO, OF THF CODE OF PUBLIC GENERAL LAWS, ENTITLED "DENTISTRY."

SECTION 1. Be it enacted by the General Assembly of Maryland, That Article 32, of the Code of Public General Laws of Maryland, entitled "Dentistry," be and the same is hereby repealed and re-enacted so as to read as follows:

SEC. 1. That from and after July 1, 1894, it shall be unlawful for any person to practice dentistry in the State of Maryland unless he or she shall have obtained a certificate of Registration, as hereinafter provided in this Article.

SEC. 2. The Governor of the State shall appoint on or before the first day of May, 1894, seven (7) persons previously recommended to him by the Maryland State Dental Association in meeting assembled, to be known as the Board of Dental Examiners, of which number two (2) shall be representatives of dental colleges or dental departments of colleges or universities chartered by the State of Maryland, and of whom one shall be from the Eastern and one from the Western shore counties of the State. The term for which the members of said board shall hold their office shall be four years, except that three members of the board first to be appointed under this Act shall be designated by the Governor to hold their offices for the term of two (2) years, and the other four (4) for the term of four (4) years, respectively, unless sooner removed by the Governor, or until their successors shall be duly appointed. In case of a vacancy occurring in said board such vacancy shall be filled by appointment of the Governor. No examiner shall serve to exceed two terms in succession. In the event of any member of said board absenting himself from two of its regular meetings consecutively, without a reasonable excuse therefor, the board shall declare a vacancy to exist, which vacancy shall be filled by appointment of the Governor.

SEC. 3. Said board shall choose one of its members president and one secretary thereof; it shall meet in May, of each year, in Baltimore city, and at such other times as it shall designate, for the purpose of attending to the duties prescribed by this Article, and shall give notice of every meeting to be held for examinations pursuant to the provisions of this Article, by advertising the place of meetings for two weeks successively in two of the daily (morning) newspapers published in the city of Baltimore, before the date of such meeting; a majority of said board shall at all times constitute a quorum, and the proceedings thereof shall at all reasonable times be open to public inspection; the board shall also make an annual report of its proceedings to the Maryland State Dental Association, and to the Governor.

SEC. 4. Every person practicing dentistry in the State of Maryland, at the time of the passage of this Act, shall be entitled to be registered by the Board of Dental Examiners, hereby created, and to a certificate of registration entitling him or her to practice dentistry within the State, upon application in writing to said board, which application shall be under oath and shall state either that said applicant has been engaged in the practice of dentistry in this State for the space of eight (8) years prior to the passage of this Act, or that said applicant holds a diploma from some dental college or university of good standing, as approved by the said board, and upon being satisfied of the truth of the statement contained in the application, and upon the receipt of a fee of one dollar, said board shall register such person in a book to be kept for that purpose, and shall issue a certificate of such registration signed by all members of said board, under the corporate seal of said board, and a copy of any registration under the hands of the president and secretary and under the seal of said board, shall be legal evidence in any of the courts of this State to prove such registration.

SEC. 5. Any person desiring to commence the practice of dentistry in this State after the passage of this Act, shall tender a fee of ten dollars and make application, under oath, to the Board of Dental Examiners, which application shall state the applicant is over twenty-one years of age, and of what Dental College or University, if any, said applicant is a graduate, and thereupon said Board shall determine whether or not to require said applicant to submit to a further examination, and if it shall be determined by the Board that said applicant shall be further examined, said applicant shall be examined under equal and fair rules and conditions, to be prescribed by said board, in anatomy, physiology, chemistry, materia medica, therapeutics, metallurgy, histology, pathology, operative and mechanical dentistry, and shall also give demonstration of his or her skill in operative and mechanical dentistry, and upon being satisfied that said applicant possesses competent knowledge and skill to practice dentistry, said board shall register said applicant and shall issue to him under the hands of all members of the board and under the seal of the board a certificate of such registration, but if said board shall determine, upon examination, that said applicant is not competent to practice dentistry, said board shall refuse to register said applicant and shall return ten dollars out of the fee previously tendered by said applicant.

SEC. 6. Any person shall be construed to be practicing dentistry within the meaning of this Act who shall, for a fee or salary or other reward, paid either to himself or to another person, treat diseases or lesions of the human teeth or jaws, or correct malposition thereof, but nothing in this Article contained shall be taken to apply to acts of *bona fide* students of dentistry done in pursuit of clinical advantages under the direct supervision of a preceptor, demonstrator, licensed dentist in this State, during the period of the enrollment in a Dental College, and while in attendance upon a regular uninterrupted course in such college.

SEC. 7. Any person who shall violate any of the provisions of this Act shall be deemed guilty of a misdemeanor, and upon conviction thereof in any Court having criminal jurisdiction shall be fined net less than ten dollars nor more than three hundred dollars, or be confined not more than six months in the county jail, in the discretion of the court for such offense; all fines received under this Article shall be paid into the public school fund of the city or county in which such conviction takes place.

SEC. 8. Nothing in this Article shall be construed so as to interfere with the rights and privileges of resident physicians or surgeons in the discharge of their professional duties.

SEC. 9. The Board of State Dental Examiners in office up to the time when this Act shall go into effect, shall turn over and account for all property of said board to the new Board of Dental Examiners which shall be appointed under the terms of this Act.

Be it enacted that St. Mary's and Calvert Counties be exempted from the provisions of this Act.

SEC. 2. Be it enacted, That this Act shall take effect from the date of its passage.

